# “Losing access to outdoor spaces was the biggest challenge for children to be healthy”: pandemic restrictions and community supports for children’s movement in Nova Scotia

**DOI:** 10.3389/fpubh.2024.1415626

**Published:** 2024-08-07

**Authors:** Maggie Locke, Becky Feicht, Michelle R. Stone, Emily Burke, Laurene Rehman, Sara F. L. Kirk, Guy Faulkner, Sarah A. Moore

**Affiliations:** ^1^School of Health and Human Performance, Dalhousie University, Halifax, NS, Canada; ^2^Healthy Populations Institute, Dalhousie University, Halifax, NS, Canada; ^3^School of Kinesiology, University of British Columbia Vancouver, Vancouver, BC, Canada; ^4^Department of Pediatrics, Dalhousie University, Halifax, NS, Canada

**Keywords:** pediatrics, neighborhoods, physical activity, play, sport, active transportation

## Abstract

**Introduction:**

Health-focused communities can promote physical activity for children by providing them with safe and supportive environments to move. Across the COVID-19 pandemic many community spaces and services were closed due to public health restrictions. During the pandemic, Atlantic Canada uniquely implemented an agreement between four provinces to restrict travel and reduce the spread of the virus. The “Atlantic bubble” led to fewer cases of COVID-19 and restrictions to community spaces and services. With restrictions now removed, community spaces and services likely play a critical role in facilitating the recalibration of children’s movement. Perspectives from families who experienced the “Atlantic bubble” may offer valuable insights to the use of these spaces during and after the removal of restrictions.

**Objective:**

This study explored the role of community spaces and services on their child’s physical activity across the COVID-19 pandemic from the perspectives of Nova Scotia caregivers.

**Methods:**

We employed a qualitative description approach and conducted semi-structured interviews with 14 caregivers of children aged 5–11 years who lived in Nova Scotia, Canada. Interviews were transcribed verbatim and analyzed using reflexive thematic analysis.

**Results:**

Four themes were generated: (1) Public health restrictions limited community movement behaviors and social connections, (2) Spaces, locations, and environments influenced how families experienced physical activity during public health restrictions, (3) Virtual realities: screens supported a new sense of community for children throughout the pandemic, and (4) “Facilitated” and “forced adaptability”: public health restrictions changed family dynamics, routines, and movement behaviors.

**Conclusion:**

Despite living in the “Atlantic bubble,” Nova Scotian caregivers shared that COVID-19 related public health restrictions shifted their family’s dynamics, routines, and ability to engage in physical activity within their communities. Community spaces and services can be leveraged to recalibrate children’s movement as pandemic-related restrictions are reduced. In future public health crises, community spaces and services should remain in place to whatever extent possible to reduce the collateral consequences of public health restrictions on children’s health.

## 1 Introduction

Ample physical activity fosters physical and psychosocial health benefits during childhood (1). The “Canadian 24-Hour Movement Guidelines for Children and Youth” recommends that all children ages 5–17 years engage in a minimum of 60 min of moderate-to-vigorous physical activity each day to achieve optimal health outcomes (1). Factors that influence a child’s physical activity can be conceptualized and interpreted using a socioecological lens (2–7). Using this framework builds understanding of the interrelationship between the child and their environments and helps identify opportunities to promote physical activity by recognizing individual and environmental factors that may encourage a child to be more active (8). Healthy communities can help promote physical activity for children by providing them with safe and supportive environments, such as schools, parks, playgrounds, and facilities where they can engage in movement (9). The healthy communities approach emerged from the World Health Organization’s Ottawa Chapter (10). A healthy community is one that protects and improves the quality of life for its citizens, promotes healthy behaviors and minimizes hazards for its residents, and preserves the natural environment” (11, p. 1500). These neighborhoods can support healthy movement for children through play-friendly community spaces (such as physical environments conducive to play, like schools, parks, playgrounds, and facilities) and supports (such as recreational and leisure-based programs offered by the community) which promote and facilitate physical activity (12, 13). In Canada, many national and local strategies to improve physical activity have focused on creating play-friendly spaces and places in neighborhoods, including designing spaces for movement and creating supportive recreation and leisure programming (14).

In March 2020, the World Health Organization declared the COVID-19 virus outbreak a global pandemic (15). Shortly thereafter, Canada responded with policies and guidelines to reduce the spread of the virus, including physical distancing, mask mandates, closures of public schools and playgrounds, and cessation of recreational and leisure programs (16). Public health restrictions varied by region and over time (17, 18). Much like other regions in Canada, Atlantic Canada subscribed to heath emergency mandates directed by the Government of Canada’s Chief Public Health Officer (19).

During the first wave of the pandemic in Nova Scotia (spring 2020), the province declared a state of emergency (20). At this time, community supports, spaces, and services were closed including schools and recreation centers (21). Throughout summer and early fall of 2020 daily case counts began to drop in Nova Scotia, allowing for public spaces including parks, playgrounds, trails, beaches, and campgrounds to reopen, as well as community recreation and leisure services and programs to restart (18, 22). However, by spring 2021, community spaces and supports were closed once again due to an increase in virus transmission. The Nova Scotia Public Health Officer further announced it was extending its ongoing state of emergency and implemented the “Atlantic bubble,” an agreement between the four Atlantic provinces to reduce travel into their provinces and reduce the spread of the virus (21). Resultantly, Atlantic Canadians tended to experience the pandemic differently. Nova Scotia’s coastal geography, low population density, and committed community response fostered world-acknowledged success in weathering the pandemic (21). The pandemic continued into 2022; however, fewer people were being infected with COVID-19 (23). With increased immunity, fewer deaths, and less pressure on hospitals, the World Health Organization finally downgraded the COVID-19 pandemic on May 5, 2023, and later that month, Nova Scotia lifted the COVID-19 health orders and ceased weekly case reporting (24). The health and economic consequences of the pandemic were unquestionable (25), and the collateral consequences of the COVID-19 related public health restrictions on physical activity of children remain an important concern. Given the Atlantic bubble and Nova Scotia’s approach to the pandemic, including keeping community supports, spaces, and services open wherever possible, Nova Scotian families may provide unique insights into the role of community supports, spaces, and services on their child’s physical activity across the COVID-19 pandemic.

Several studies have illustrated the negative impacts of pandemic-related restrictions on physical activity among children (22, 26–31). Overall, children spent less time engaging in physical activity throughout the pandemic compared to before the pandemic, and fewer children met movement-related recommendations (26, 27). Studies attributed closures of spaces and cessation of programs to the lower proportion of children meeting physical activity guidelines, and caregivers expressed their frustrations about changes in community supports and spaces (32). Varying public health restrictions across regions in Canada impacted opportunities for children’s movement; regions with higher number of cases of COVID-19 and the most stringent restrictions on access to the outdoors had greater declines in outdoor play (17), and children within these regions were also less likely to meet movement behavior guidelines compared to regions with fewer cases and less restrictions (29).

With generally fewer cases of COVID-19 and resultant public health restrictions in Atlantic Canada, children’s physical activity seemed to be less impacted than other parts of the country (22, 29). This may have been due to Nova Scotia’s strict response at the outset of the pandemic, including the closure of community spaces, supports and services. The province enforced these restrictions by handing out hefty fines for those visiting outdoor community spaces such as parks and beaches due to violation of the emergency measures act (33). However, public health restrictions were relaxed as fewer cases of COVID-19 were reported compared to the rest of the country (34). The Atlantic bubble saw Nova Scotia joining with other Atlantic provinces (New Brunswick, Prince Edward Island, and Newfoundland and Labrador) to allow for increased travel throughout only these provinces. Children in Nova Scotia soon returned to community spaces and services, though still with restrictions on registration numbers compared to pre-pandemic, as the Atlantic bubble was implemented (35). Data is not available specific to Nova Scotia immediately prior to the pandemic; however, prior to the onset of the COVID-19 pandemic and associated public health restrictions, approximately 17.5% of Canadian children and youth were meeting the 24-h movement guidelines (36). According to the 2018 Canadian ParticipACTION report card, prior to the COVID-19 pandemic, 77% of Canadian children and youth were participating in organized sport, 20% of children and 37% of youth engaged in more than 2 h of active play outside of school, and 36% of Canadian children and youth engaged in active family play or activities (37). In Canada, initial studies on the pandemic’s influence on children’s physical activity showed that only 1% of children were meeting the 24-h movement behavior guidelines during the height of the pandemic; whereas 1 year later with loosened restrictions, this increased to 7% of children meeting recommendations (22). Canadian caregivers reported that having access to community spaces to be active throughout the pandemic helped facilitate their child’s movement; however, most children were still not meeting recommendations despite loosening restrictions (22).

Given the unique experiences of families living in the Atlantic bubble during the pandemic, it would be valuable to understand how the pandemic and its related public health restrictions impacted children’s physical activity in Nova Scotia, and family’s perspectives on the role of community spaces and services to support movement. While some studies have explored caregiver (32) or child perspectives (38), these studies have not explored the experience in Nova Scotia, where the Atlantic bubble presented different approaches to restrictions of community spaces and services. Studies from other parts of the country identified caregiver concern about decreased social opportunities at school and after school programs (38), difficulties accessing organized community services (e.g., sports programs) and disrupted routines (39, 40) resulting from COVID-19 public health restrictions, but it is not clear whether these experiences were shared by Nova Scotian caregivers. Overall, Canadian caregivers have associated public health restrictions negatively with their child’s physical and mental health and well-being (while understanding their necessity) (39, 41). Nova Scotian caregivers may have different perspectives as public health officials were supportive of families being outdoors in community spaces (while maintaining distancing) and several local community organizations maintained programs and services (while still implementing other measures to reduce virus transmission, such as masking) (42). Community environmental characteristics, such as proximity to parks and other community services, also influenced children’s opportunities for movement in Canada (28). In Nova Scotia, closer proximity to parks and community services was found to positively influence children’s activity (22), so it may be worthy to ask caregivers about their experiences in accessing these spaces. We do know that Canadian caregivers were concerned about virus transmission, which may have influenced children’s social freedom and independent mobility within their communities (43). Fewer children were engaged in active transportation during the pandemic (44, 45). Canadian caregivers also reported struggling to understand public health restrictions and were having difficulty adapting to the changing public health restrictions; resultantly, their children were engaging in more screen-based activities and reduced physical activity (32). Canadian caregivers also noted their reliance on community programs for their children’s physical activity, but noted that these options cessed during COVID-19 (32). Whether these experiences were the same in Nova Scotia has yet to be examined. With the Atlantic bubble in place, fewer cases of COVID-19, and fewer health restrictions, the unique perspectives of Nova Scotian caregivers may provide valuable insights as to how community spaces and services may be utilized to keep children moving during public health crises.

Therefore, this study explored the role of community spaces and services on their child’s physical activity across the COVID-19 pandemic from the perspectives of Nova Scotia caregivers.

## 2 Methods

### 2.1 Study design

This study used a qualitative description design to explore gain an understanding of participant perspectives and describe participants’ lived experience (46). We utilized the COnsolidated criteria for REporting Qualitative research (COREQ) checklist (see Supplementary file S1). Semi-structured interviews were conducted with caregivers of children ages 5–11 years in Nova Scotia. During the interviews, caregivers were asked to describe their child’s and family’s experience throughout the COVID-19 pandemic, the factors influencing physical activity in the context of public health restrictions, and the role of community spaces, supports, and services in supporting children’s movement across the pandemic (see Supplementary file S2). This study was reviewed by Dalhousie University’s Research Ethics Board (#2022-6040).

### 2.2 Participants and recruitment

This study used a non-probabilistic purposive sampling technique from within a larger national sample of Canadian families of children who participated in a quantitative survey assessing changes in children’s movement behaviors during the pandemic (47). Nova Scotian caregivers who completed this quantitative survey and expressed interest in participating in an interview were recruited to participate in this study. Purposeful sampling is widely used in qualitative research as it allows researchers to identify and to select a rich variety of participants that fit within the inclusion criteria to represent multiple realities (48). Paterson et al. (45) collected data for a larger study examining the changes in parenting practices over the course of the pandemic from three provinces (Nova Scotia, Ontario, and British Columbia). A sub-sample of this data was used for the purpose of this research. Participants were eligible to participate in this study if they met the following inclusion criteria: (a) being a parent or guardian (“caregiver”) of a child ages 5–11 years; (b) living in Nova Scotia; (c) able to understand and communicate in English; (d) having access to a computer with a reliable internet connection for a virtual meeting; and (e) no pre-existing relationships between the researchers and caregivers.

### 2.3 Data collection

Semi-structured one-on-one interviews were conducted virtually with participants through the online platform, Zoom, in June and July 2021. Participants were informed about the study’s purpose and provided informed consent prior to the interviews. Semi-structured and open-ended questions were used to encourage participants to express themselves freely (49). All interviews were conducted by the same student research assistant (DP) and were 60–90 min in length. Student was male and held MA credential. Interviews were audio recorded to be transcribed manually and verbatim.

### 2.4 Data analysis

Data were analyzed using reflexive thematic analysis informed by Braun and Clarke (50–52). Reflexive thematic analysis is a way to explore patterns and develop themes in qualitative data, while remaining critical and reflective of our role as researchers. Our analysis process involved a team led with the support of research assistants, who had backgrounds in health promotion, recreation and leisure, or exercise science. Our team began with considering and reflecting upon our own positions and how they shape our views, broadly, and more specifically regarding our research purpose. Our researchers valued physical activity and outdoor play and they experienced their own challenges with engaging in activity during the COVID-19 pandemic. All researchers (apart from GF) lived in Nova Scotia and had experienced the changing public restrictions in the Atlantic bubble. Researchers who conducted these analyses did not have first-hand experience as caregivers, but findings were reviewed and interpreted by other members of the team who had these experiences. The team gained familiarity with the data by reading and re-reading the transcripts, taking notes, and reflecting upon how their values and experiences may have shaped their views regarding this research. Weekly meetings with the research team provided the opportunity to discuss our thoughts as familiarity with the data developed. We completed inductive coding using NVivo software, developing both latent and semantic codes and systematically tagging segments of transcripts with what we determined to be meaningful to our research questions. We began coding collaboratively, to allow reflection and discussion, and progressed to independent coding once familiarity and comfort with the dataset had developed. We used a shared codebook that was created and iteratively updated collaboratively. Themes were then developed by examining how codes might fit together to form patterns of shared meaning. Before finalizing themes, the findings were discussed and refined by the research team. This process allowed the results to be woven into a narrative reflecting a socioecological lens.

## 3 Results

A total of 14 interviews were conducted with Nova Scotian caregivers of children ages 5–11 years. Eleven caregivers identified as mothers, and three identified as fathers. Caregivers reported on their child(ren)’s age and gender, identifying eight boys and six girls between the ages of 5–11 years with a mean age of 9.7 years. Other demographic information was collected from participants and can be viewed in detail in the Supplementary file S3.

Four themes were developed using reflexive thematic analysis. The first theme explores how public health restrictions limited community movement behaviors and social connections; the second theme describes how spaces, locations, and environments are essential to how “community” were experienced during the COVID-19 pandemic and public health restrictions (i.e., the social bonds within and between people and places); the third theme examines “virtual realities” that were created and how public health restrictions limited community engagement, drawing children to screens; and the fourth theme describes how caregivers perceived COVID-19 public health restrictions to changed family dynamics, routines, and the engagement of their child in movement.

### 3.1 Theme 1: public health restrictions limited community movement behaviors and social connections

Caregivers agreed that public health restrictions impacted their child’s engagement in community spaces and programs, and access to resources, which made it more difficult for their child to be physically active. One caregiver discussed “*everything being closed*” and described how their children wanted to access community recreation opportunities, but could not, due to public health restrictions. They said, “…*they wanted to play hockey and they could not. They wanted to go to the ski hill but could not*” (NS10). Another participant said, for example, “*We went from five extra-curricular activities throughout the week to nothing. Everything was gone*…” (NS12). An additional participant remarked, “*practicing in the backyard yea ‘cause it was like the batting cage was closed and the baseball field was closed and everything was pretty much closed*” (NS9).

Caregivers said they needed to navigate public health restrictions, but the stories from caregivers were influenced by each family’s comfort levels with risk of infection. One participant reflected on their own cautiousness, saying, “*I would say our comfort level changed with the active cases […] we are more cautious than most*” (NS10). Another caregiver said, “*It was interesting because, uh you know, we have a little chat group […] one parent reached out, ‘How does everybody feel? Are you ok if the kids go out to play? My kids are going to wear masks’*” (NS6). Overall, there was a common perception that the cessation or changes to programs or the closure of spaces left families responsible for navigating public health restrictions and finding opportunities for their children to participate in physical activities.

Public health restrictions also created barriers in connecting with others, limiting opportunities for children to engage in social play. Many participants discussed the isolation of the pandemic, like one caregiver who commented that a challenge for their child was “*being alone, secluded to your home and not having the connections to friends*” (NS 12). Other caregivers noted how “*we had to keep our bubble small*” (NS16) and “*we kept ourselves isolated*” (NS3). Several participants noted the important social aspects of play, recreation, and physical activity, especially for their children, and reflected on the associated challenges with public health restrictions. One said, for example, “*during the lockdown they were not allowed to play with one another*” (NS15), and another said, “*you could not play with people*” (NS1). Many caregivers identified the importance of connecting socially for well-being, and how that was made challenging by public health restrictions.

### 3.2 Theme 2: spaces, locations, and environments influenced how families experienced physical activity during public health restrictions

Through our analysis of the caregiver interviews, it was evident how spaces, locations, and environments contributed to feelings of a “community” and how sense of community was affected with the public health restrictions. Here, community is characterized more broadly than the physical environment where people live and includes the social bonds between people and places. Caregivers also discussed the important influence of space and place, and factors like urbanity and rurality, as interconnected with public health restrictions and their child’s movement behaviors. One caregiver said, for example, “*Yeah, our farm we have 55 acres. We are along a nice river. We are probably out more than we are in*” (NS3), indicating that living rurally helped to support their child’s physical activity during public health restrictions. In the first theme, it was apparent that caregivers saw public health restrictions change the way they were accessing community resources and programs. Now in the second theme, caregivers also noted changes in their child’s unstructured activities as well, like outdoor play, some positively, and others less so. This was particularly relevant based on the family’s rural or urban living. One caregiver said, “*we were blessed living on a lake and everyday going swimming. They have a water trampoline. They get their exercise when they are out on the water*” (NS10). Another described accessing more rural areas for activity, saying, “*we spent a lot of time at either my boss’s house because they live in the country […] or my grandmother’s house where they could just run free and walk in the woods and trails and beach*” (NS16).

Over time, as restrictions lessened, and with transitions in the weather, caregivers noted a change in their children’s physical activity behaviors. Some described encouraging their children to be active through public health restrictions to be “*a struggle*,” but noted that when the weather warmed, it was easier, saying, “*during the summer […] the weather is a little nicer, there’s been more opportunity to get outside and do things*” (NS16). Another caregiver discussed this seasonal contribution to their child’s activity as well by saying, “*Summer’s been good. We’ve been very active. We camp, we hike, we go to the beach. We go biking, kayaking, go for walks, we are always on the go*” (NS15). Others noted the challenges of the winter season, saying, “*the winter was tougher in general. The kids were not as motivated to get outside. Being inside together there was more arguments between them*” (NS3). Overall, caregivers noted the interplay between changing COVID-19 cases, public health restrictions, seasonal shifts, and urban versus rural living. There was a temporal rhythm to the stories provided, with lower child activity noted and more struggle when COVID-19 cases and related public health restrictions were high, or when weather was cold, compared to when cases and restrictions eased, weather was warm, and rural space was available for movement.

A further impact of public health restrictions earlier in the pandemic was the need for families to “*really just stay close to home*” (NS8) and seek opportunities for physical activity nearby. One caregiver described early lockdown periods, saying, “*when things are in lockdown, there are less places to go. Even though we had ski passes, you could not leave to ski […] You sign them up for a program, then the program gets canceled*” (NS4). Another said, similarly, “*A lot of the beaches you were not allowed to go to. Not allowed to go to parks. The places the kids were familiar with we could not visit every day like we used to*” (NS11). For some caregivers, there was an acknowledgement of privilege (income, housing, affordability, choice). One caregiver noted, for example, “*We live on a lake, so we are blessed with that ability… to swim, go for a boat ride or a canoe*” (NS10), and acknowledged the benefit (for their family) of living more rurally during public health restrictions; while another acknowledged the challenges of apartment living, saying, “*just trying to keep him active really and just trying to you know uh, we live in an apartment so the space is small*” (NS5). Overall, the stories we heard from caregivers demonstrated that different community types, spaces, and places afforded families different experiences in unstructured play, physical activity, and movement, and while restrictions were high, their “community” spaces were impacted.

### 3.3 Theme 3: virtual realities: “screens supported a new sense of community for children throughout the pandemic”

As described in previous themes, public health restrictions sometimes limited children and family’s engagement with their built environments, as well as access to programs, services, and recreation opportunities. Less structured and unstructured physical activity in and about a child’s community were perceived to influence children’s screentime. One caregiver said, “*the restrictions coming and going, and everyone has been on electronics more*” and “*more time on electronics because what else is there to do?*” (NS1). Another caregiver summarized this well, saying, “*Their screen time is up because other things are down*” (NS4), indicating the relationship between closure of spaces and cessation of programs (public health restrictions) and screen time.

The increase in screen time related to public health restrictions also intersected with online learning environments and a need for social connections in the early parts of the pandemic for Nova Scotian families. Several caregivers noted the early “*switch to the online learning*” (NS8), and caregivers expanding roles, where they “*had to be on at certain times to see the teachers and to do the work*” (NS1). Many caregivers noted that due to public health restrictions children had limited access to physical (built environment) community spaces to engage with their peers, and therefore, turned to screen time for those connections. One caregiver described their child communicated “*through his tablet to chat with friends*” (NS11), while another noted their child enjoyed the opportunity to “*play online with his friends*” (NS5). Another caregiver described the value of screen time in allowing their child to connect with friends during the highest public health restrictions, saying, “*When he is on the electronics playing games, he is able to talk to his friends so there is that bit of connection when he could not see them in person*” (NS1).

Another important intersection of public health restrictions and screen time related to the need for caregivers to balance multiple demands. One participant described this shift in routine by saying, “*Normally we would get up and I would go to work, and she would go to school. So, I guess that time was filled instead with media*” (NS3). Other caregivers highlighted how challenging balancing multiple demands and limiting screen time can be, saying, “*when you work you can only do so much*” (NS4) and “*I know [it] is not great for him to be on the screen time as much but [it] is kind of just like you are exhausted*” (NS1). Another caregiver described shifting views on screen time, saying, “*I did what I swore I would never do, and I let my kids play video games and I let them play way too much*” (NS7). Another summarized this well, noting that allowing children to use screen time allowed them to “*occupy themselves […] and that […] allow[ed] me to focus on the things I need[ed] to do*” (NS4). Caregivers noted the change over time to their child’s screentime. Earlier in the pandemic, screentime was highest when restrictions were strict, whereas children began to return to play and opportunities in their communities, screentime seemed to decrease. Screens provided a redefined sense of community for families (connecting online with friends and family) when they were forced inside in the early parts of the pandemic. There was a collective note of relief from caregivers when restrictions were loosened, and social connections could move from the virtual to their child’s physical community.

### 3.4 Theme 4: facilitated and forced adaptability: public health restrictions changed family dynamics, routines, and movement behaviors

The final theme highlights how families adapted when faced with public health restrictions, and the corresponding impact on children’s movement behaviors. We label this adaptability as both “facilitated” and “forced” as the caregivers in our study discussed the significant impact of public health restrictions on their family dynamics, routines, and their children’s movement behaviors, with both positive and negative tones and perceptions. The themes presented above demonstrate several examples where caregivers discussed how public health restrictions limited how families engaged with their community. Certainly, families were forced to adapt, and this brought about challenges for some caregivers in helping their children participate in healthy activities. For example, one caregiver noted that “*losing access to some of these outdoor spaces was the biggest challenge to provid[ing] opportunities for the children to be healthy*” (NS11). These challenges were exacerbated by the family’s changing routine. Several caregivers noted that the change in routine, with their child disengaging in community-based programs and services, was the biggest challenge they faced.

The change in routines due to public health restrictions was significant for families, particularly during the early parts of the pandemic, and contributed not only to a change in schedules, but also to family dynamics. While this was difficult at first, many caregivers responded to our questions about this with optimism and positivity, by saying, for example: “*I certainly spend more time with them*” (NS4); “*the time spent together […] just bonding*” (NS5); and “*in ways it brought us closer*” (NS16). One caregiver noted a shift in roles, saying, “*I am not just mom, I am their friend, and I have to kind of step in for grandmother and aunt and everyone they used to see […] I just tried to keep things consistent for them*” (NS3). Many caregivers noted the shift in routine, facilitated activities which brought the families together, for example, “*Everyone was home and doing more electronics than they should have. But we are pivoting and trying to do things together at the same time*” (NS1).

Caregivers directly linked the shift in family dynamics, routines, and activity to the increased public health restrictions. One caregiver said, “*When there is a lockdown and there is nothing else to do, we went on walks. When you are told to just stay home, we walked more as a family […] my younger daughter and my husband would go on bike rides*” (NS4). Overall, caregivers took to the task of recalibrating their family’s routine. For example, several caregivers described the need “*to be more creative*” (NS10), and that this creativity continued throughout the pandemic, even when the restrictions loosened. One caregiver said, for example, “*what can we do today? Let us go on a scavenger hunt, or let us go on a hike. Let us take the dogs somewhere […] something…*” (NS15). Another appreciated that this creativity and change in routine also brought the family together, “*we had to do things with our own families, so we were doing things together more*” (NS1). It seemed common that public health restrictions facilitated an increase in family physical activity. While most caregivers focused their responses on the earlier parts of the pandemic when restrictions were loosened, many noted the forced change was taken up as a challenge and resulted in a creative approach to family time and included engaging as a family in physical activities.

While the COVID-19 pandemic and public health restrictions brought about incredible challenges for families, the disruptions also provided opportunity for adaptations in how families engaged in physical activity and utilized resources in their communities. We heard stories of adaptability, resilience, and optimism. Caregivers approached reduced access to community programs and services with flexibility, saying, for example “*We just have to play more sports and get them into different activities […] It sucks but keep it going […] We just adapted*” (NS13). Some caregivers said that their children were even more resilient, saying, “*They adapt well, children, I find. They adapt easier than we do, I think*” (NS8). Finally, one caregiver said, regarding family-wide physical activity and the shift in family dynamics, “*it was a bright light in the pandemic*” (NS1). They also learned to live differently during the pandemic. Caregivers missed the access they had to community spaces, supports, and services, but the resulting shifts to more family time were welcomed. As caregivers spoke about their child’s return to community with lessened restrictions, they hoped to strike a balance which would keep families connecting while they re-engaged in their communities.

## 4 Discussion

The purpose of this study was to explore caregivers’ perceptions of community spaces, supports, and services, that are connected to the movement behaviors of their children during the COVID-19 pandemic and associated public health restrictions in Nova Scotia. Themes were developed based on families’ experiences throughout the pandemic. The stories we heard from caregivers indicated that they initially felt unsupported, yet they struggled to navigate the pandemic’s public health restrictions and keep their children active. The caregiver stories indicated that public health restrictions affected children’s movement behaviors, and this differed across locations and time periods as it related to varying public health restrictions. The perspectives shared allowed us to see the perceived contributions of spaces, locations, and environments to children’s physical activity. Caregivers often noted how public health restrictions impacted children’s social connections within their communities and resulted in increased social connections through screentime. An increase in screentime often displaced opportunities for movement, particularly in the early parts of the pandemic when the restrictions were at an all-time high. Overall, the stories we heard drew attention to the impact of public health restrictions on families’ dynamics, routines, and in turn, their engagement in physical activity. Children and their families were forced to adapt to the ongoing, changing public health restrictions. While caregivers found these adaptations incredibly challenging, the stories we heard indicated that many families saw this time as an opportunity to spend more time together, engaging and moving together.

Using a socioecological lens, the results from this study illustrate various community and individual level factors that have influenced children’s movement behaviors across the pandemic with changing public health restrictions in Nova Scotia. In addition, community level factors, such as the closure of community spaces, supports, and services influenced individual health as public health restrictions dictated children’s environments and movement behaviors. While the Nova Scotia COVID-19 landscape was seemingly different during the pandemic (given lower cases of COVID-19, fewer restrictions, and the implementation of the Atlantic bubble), these stories did not seem to be particularly unique to our geographic region. However, attention was drawn to the impact of rural living and how this helped facilitate movement when community spaces, supports, and services were not available. Despite the interview prompting for stories across the entire duration of the pandemic, most caregivers centered stories around the most challenging times, when restrictions were highest, although collectively we heard a sense of relief for less COVID-19 infections in the province, the loosening of public health restrictions, and renewed availability of community spaces, supports, and services.

Access to community spaces, supports, and services are known to contribute to children’s physical and psychosocial health (53). Communities can help promote physical activity by enabling access to community spaces, supports, and services where people can engage in movement behaviors (12). Public health restrictions put in place due to the COVID-19 virus have affected families access to their communities and associated services (32). In Nova Scotia, we saw initial public health restrictions result in the closure of schools, parks and playgrounds, and cessation of community programs such as sports; resultantly, children disengaged from these community spaces, supports, and services, and there was a notable decline in physical activity (28). Caregivers in our study frequently reflected on this early part of the pandemic, and noted how the closure of schools and community programs limited their engagement with their community and created (sometimes unnecessary) barriers to physical activity for their children. There is a need to consider ways to balance restrictions that support reduced virus transmission, while supporting children’s opportunity to engage in physical activity (54). Given the known health benefits of engaging with, and moving in one’s community (53), it is not as simple as closing community spaces, supports, and services. For future health crises that require public health restrictions it is important that public health professionals and policy makers find solutions that will allow for children to continue to engage and have access to their communities supporting their physical activity while keeping them as safe as possible to support their health.

Research prior to the pandemic supports the notion that rural families rely more on natural features in their surroundings, such as woods and open fields, as places where their children can be physically active, participating in unstructured activities, compared to urban families, who may rely more on structured activities in their communities (55). Interestingly, these features played an important role for the children of caregivers in our study who lived in rural settings, as during the pandemic they had more open outdoor space which they could access to play and engage in family physical activity. In urban communities, play-friendly designs can support children’s movement. For example, safe, active, and accessible routes can contribute to improved movement in community spaces (56). In response to public health restrictions, cities all over Canada, including in Nova Scotia, adapted to keep their community members active during the COVID-19 pandemic (57). For example, the Halifax Mobility Response Plan was put in place May 2020 with the aim to implement short, medium, and long-term action to public spaces including slow streets, sidewalk expansions, and street closures to promote movement behaviors while abiding by the public health restrictions (58). These efforts attempted to support movement while observing important physical distancing measures. However, with strong messaging about “staying home” during the pandemic, Nova Scotian children and families may not have been able to fully take advantage of these changing and potentially more play-friendly streetscapes. Coordinating these efforts in future health crises may allow more children to access their play-friendly communities while still adhering to necessary public health mandates. A recent content analysis of Nova Scotian municipal policy documents related to playability indicated that many communities in Nova Scotia have action plans to support more physical activity; however, policies and strategies varied from community to community and were not consistently implemented (28). Establishing and implementing play-friendly communities may be one way to recalibrate children’s movement in the era of COVID-19, but also support continued access to places to move in the event of future health emergencies.

A child’s neighborhood environment is a key factor that contributes to engagement in physical activity (59). Most research (pre-COVID-19) shows that higher dwelling density communities, such as larger sized cities with more mixed land use have infrastructure to support and enable children’s active transportation (i.e., walking, wheeling, biking), and easy/connected access to community-based spaces, such as sport and recreation facilities (28). The pandemic related public health restriction affected how children experience outdoor play in their neighborhood (60). Unlike previous research, the caregivers in our studies shared that those with homes with more space (e.g., acreage, rural living) allowed for their children to engage in more physical activity during the pandemic, as they could use their larger outdoor spaces to engage in movement when public health restrictions kept them home. For caregivers in our study who lived in more urban settings, in apartment buildings, or who had limited outdoor space on their property, there was a disengagement in outdoor physical activities. This may also be linked more directly to factors of socioeconomic status rather than simply environmental factors, given that caregivers who lived in rural environments described using funds to create spaces for their children to play during the pandemic (adding a pool, basketball court). Not surprisingly, weather also played a role, where warmer drier weather resulted in more opportunities for children to engage in physical activity, which was also in line with when cases of COVID-19 dropped in Nova Scotia and restrictions loosened. It is well accepted that weather is a determinant of children’s physical activity and that in most North American and Western European countries, warmer drier weather is associated with more movement (61).

Caregivers noted their typical reliance on structured activities for their children’s physical activity, and when these opportunities were not available, it caused stress. Other studies have also described caregiver reliance on structured programming for their children’s physical activity (62). In our study, there seemed to be a shift that occurred when these structured opportunities for children’s physical activity were no longer available. Many of the caregivers in our study reported that the cancelation of community services, for example, sport practices or recreational programs initially negatively affected their child’s movement behaviors and then forced them to rely on more unstructured activities at home compared to before the COVID-19 pandemic. Caregivers in our study told stories of breaking free from structured programming and enjoying physical activity at a more leisured pace. This was a welcome change for many families. There is often a lack of opportunities for children to freely play and move about home and community spaces in unstructured ways (63). More opportunities for freely chosen and child-driven physical activity can support children’s health and well-being (64). Caregivers in our study noted their appreciation for newly found family time, and a desire to continue this as they reset and establish their new routines.

The increase in public health restrictions and the subsequent decrease in community services during the pandemic not only impacted children’s engagement with their community but they were also related to increased screen time. Caregivers in our study provided accounts of their child watching significantly more screens (TV, tablet, video games, etc.) when public health restrictions initiated stay-at-home orders and closed schools, parks, and playgrounds early in the COVID-19 pandemic. Increased screentime during the lockdown periods has been identified in several studies (65). Caregivers in our study voiced that the increase in their child’s screentime was alarming, although necessary, for their child’s socialization when public health restrictions were at their highest. Screentime was not simply for recreational use during the COVID-19 pandemic, children turned to their devices for online school, virtual community activities, and communication with their peers. Schools moved classes to online and many recreation programs offered virtual sessions in lieu of in-person opportunities (66). Transitioning to virtual options was a big pivot for community programming but seemed to be working to support children’s movement and development (67). While online options provided access to community programming for many, some community members may have been excluded due to poor internet availability in rural and remote areas (68). So, while virtual programming may be one solution to keep children moving in future lockdown scenarios, it must be remembered that there is still a significant digital divide in Nova Scotia and across Canada for those in rural and remote areas. Regardless, virtual community programming still does increase screentime, which can have negative implications for children (69). It may be worthwhile, instead, to extend efforts to connect children to their community spaces during future health crises, rather than focusing on digital solutions. Perhaps, future work can find the right balance between these options in times of health crises.

### 4.1 Strengths and limitations

To our knowledge, this is the first study to qualitatively explore the impact of the COVID-19 pandemic and associated public health restrictions on Nova Scotian children’s movement behaviors. We situated this study using a socioecological lens and examined caregiver perspectives of community spaces, supports, and services to support their child’s physical activity during the COVID-19 pandemic. We used reflexive thematic analysis which allowed researchers to reflect on their own values and positions, which was consistently, coherently and transparently enacted throughout the analytic process of reporting the research (51). This method allowed for closer examination of the lived experiences of Nova Scotian caregivers across the COVID-19 pandemic, specifically examining how community related to their children’s movement behaviors. The flexibility of reflexive thematic analysis is a strength of this methodology, as it provides theoretical freedom to allow for rich and detailed, yet complex accounts of the data, with knowledge production being at the heart of the approach (50–52).

This paper is not without its limitations. Our participants are from different geographical regions (some urban and some rural) and have children who represent diverse perspectives by age and gender. However, we note that many of our participants described experiences that would not be feasible for some families facing socioeconomic constraints. Caregivers who were struggling during the pandemic may have been less likely to respond to our recruitment and we may have missed capturing those experiences in this study. We also note that our study is limited by exclusively interviewing caregivers to assess the role of community spaces, support, and services on children’s movement. Future research should explore the perspectives of community organizations who provide such services, but more importantly the perspectives of children.

## 5 Conclusion

The COVID-19 pandemic had an undeniable impact on Nova Scotian children. Caregivers in our study described adapting their family’s routines in response to public health restrictions that limited access to community spaces, supports, and services. Caregivers noted several ways that restricted access to community impacted their children’s physical activity. The benefits of physical activity for children are well documented, and currently, most children are not being active enough to support their health. Now that COVID-19 has been downgraded and is no longer a public health emergency, public health professionals need to consider how to re-engage children in physical activity to support their health and reconnect children and their families within their communities. Important lessons have also been identified for navigating any future public health crises that could impact the movement behaviors of children.

## Data availability statement

The raw data supporting the conclusions of this article will be made available by the authors, without undue reservation.

## Ethics statement

The studies involving humans were approved by Dalhousie University Research Ethics Board (#2022-6040). The studies were conducted in accordance with the local legislation and institutional requirements. The participants provided their written informed consent to participate in this study.

## Author contributions

ML: Data curation, Formal analysis, Writing – original draft, Writing – review & editing. BF: Formal analysis, Supervision, Writing – original draft, Writing – review & editing. MS: Formal analysis, Writing – review & editing. EB: Formal analysis, Writing – review & editing. LR: Funding acquisition, Supervision, Writing – review & editing. SK: Funding acquisition, Supervision, Writing – review & editing. GF: Conceptualization, Data curation, Formal analysis, Funding acquisition, Investigation, Methodology, Project administration, Resources, Writing – review & editing. SM: Conceptualization, Data curation, Formal analysis, Funding acquisition, Investigation, Methodology, Project administration, Resources, Supervision, Writing – original draft, Writing – review & editing.
