# Supplementary material for: “Losing access to outdoor spaces was the biggest challenge for children to be healthy”: pandemic restrictions and community supports for children’s movement in Nova Scotia
Source: Front Public Health. 2024 Aug 7;12:1415626. doi: 10.3389/fpubh.2024.1415626 (PMC11335493; doi:10.3389/fpubh.2024.1415626)
Supplement: Supplementary file 3 [file Table_3.DOCX]

**Supplementary File 3: Participant Demographic Information**

| **Participant Number** | **Parent Interviewed** | **Marital Status of Parent Interviewed** | **Family’s Dwelling Type** | **Child’s Age (yrs)** | **Child’s Gender** | **Child’s Siblings (#; age (yrs); gender)** |
| --- | --- | --- | --- | --- | --- | --- |
| NS1 | Mother | Married | Detached house | 11 | Boy | One sibling: 14-years, girl |
| NS3 | Mother | Married | Detached house | 7 | Girl | One sibling: 4-years, boy |
| NS4 | Mother | Married | Detached house | 11 | Girl | One sibling: 9-years, girl |
| NS5 | Mother | Separated | Low-rise apartment | 11 | Boy | No siblings |
| NS6 | Mother | Widowed | Detached house | 10 | Boy | Two siblings: 18- and 20-years, girls |
| NS7 | Mother | Married (away) | Detached house | 8 | Boy | One sibling: 8-years, boy |
| NS8 | Mother | Married | Detached house | 11 | Girl | One sibling: 13-years, boy |
| NS9 | Father | Married | Detached house | 12 | Boy | Three siblings: 12-years, boy; 2-years and newborn (gender not reported) |
| NS10 | Father | Married | Detached house | 10 | Boy | One sibling: 8-years, boy |
| NS11 | Mother | Common law | Not reported | 8 | Girl | Two siblings: 2- and 5-years, girls |
| NS12 | Mother | Single, never married | Detached house | 9 | Girl | No siblings |
| NS14 | Father | Married | Low-rise apartment | 10 | Boy | No siblings |
| NS15 | Mother | Single, never married | Camper home | 9 | Girl | No siblings |
| NS16 | Mother | Married | Detached house | 9 | Boy | Two siblings: 7-years, girl and 3-years, boy |
